# Validation of 2D flow MRI for helical and vortical flows

**DOI:** 10.1136/openhrt-2023-002451

**Published:** 2024-03-08

**Authors:** Zia Mehmood, Hosamadin Assadi, Ciaran Grafton-Clarke, Rui Li, Gareth Matthews, Samer Alabed, Rebekah Girling, Victoria Underwood, Bahman Kasmai, Xiaodan Zhao, Fabrizio Ricci, Liang Zhong, Nay Aung, Steffen Erhard Petersen, Andrew J Swift, Vassilios S Vassiliou, João Cavalcante, Rob J van der Geest, Pankaj Garg

**Affiliations:** 1 Norfolk and Norwich University Hospitals NHS Foundation Trust, Norwich, UK; 2 Department of Cardiovascular and Metabolic Health, University of East Anglia Norwich Medical School, Norwich, UK; 3 Department of Cardiovascular and Metabolic Health, University of East Anglia, Norwich, UK; 4 Department of Infection, Immunity & Cardiovascular Disease, University of Sheffield, Sheffield, UK; 5 National Heart Centre Singapore, Singapore; 6 Neuroscience, Imaging and Clinical Sciences, Gabriele d'Annunzio University of Chieti and Pescara, Chieti Scalo, Italy; 7 Queen Mary University of London, London, UK; 8 Advanced Cardiovascular Imaging William Harvey Research Institute, The London Chest Hospital, London, UK; 9 University of Sheffield, Sheffield, UK; 10 Cardiovascular, Cleveland Clinic Foundation, Cleveland, Ohio, USA; 11 Leiden University Medical Center (LUMC), Leiden, The Netherlands

**Keywords:** Magnetic Resonance Imaging, Aortic Diseases, Heart Failure, Aortic Valve Stenosis

## Abstract

**Purpose:**

The main objective of this study was to develop two-dimensional (2D) phase contrast (PC) methods to quantify the helicity and vorticity of blood flow in the aortic root.

**Methods:**

This proof-of-concept study used four-dimensional (4D) flow cardiovascular MR (4D flow CMR) data of five healthy controls, five patients with heart failure with preserved ejection fraction and five patients with aortic stenosis (AS). A PC through-plane generated by 4D flow data was treated as a 2D PC plane and compared with the original 4D flow. Visual assessment of flow vectors was used to assess helicity and vorticity. We quantified flow displacement (FD), systolic flow reversal ratio (sFRR) and rotational angle (RA) using 2D PC.

**Results:**

For visual vortex flow presence near the inner curvature of the ascending aortic root on 4D flow CMR, sFRR demonstrated an area under the curve (AUC) of 0.955, p<0.001. A threshold of >8% for sFRR had a sensitivity of 82% and specificity of 100% for visual vortex presence. In addition, the average late systolic FD, a marker of flow eccentricity, also demonstrated an AUC of 0.909, p<0.001 for visual vortex flow. Manual systolic rotational flow angle change (ΔsRA) demonstrated excellent association with semiautomated ΔsRA (r=0.99, 95% CI 0.9907 to 0.999, p<0.001). In reproducibility testing, average systolic FD (FDsavg) showed a minimal bias at 1.28% with a high intraclass correlation coefficient (ICC=0.92). Similarly, sFRR had a minimal bias of 1.14% with an ICC of 0.96. ΔsRA demonstrated an acceptable bias of 5.72°—and an ICC of 0.99.

**Conclusion:**

2D PC flow imaging can possibly quantify blood flow helicity (ΔRA) and vorticity (FRR). These imaging biomarkers of flow helicity and vorticity demonstrate high reproducibility for clinical adoption.

**Trials registration number:**

NCT05114785.

**WHAT IS ALREADY KNOWN ON THIS TOPIC**

- Despite some of the advances in knowledge and technology for in vivo assessment, there is limited literature on assessing aortic helical and vortical flows and their impact on clinical outcomes in various cardiovascular diseases. While capable, four-dimensional (4D) flow cardiovascular MR (CMR) is intricate and time-consuming to perform and not widely available. Consequently, its potential impact to link the association of aortic flow haemodynamics with various cardiovascular diseases is restricted. Previous studies have shown the relevance of these functional parameters of aortic flow and their association with reduced aortic conduit and reservoir function.

**WHAT THIS STUDY ADDS**

- This study proposed a simplified approach for broader clinical assessment of helical and vortical flows in the aorta, which can be measured by two-dimensional (2D) phase-contrast (2D PC) CMR. 2D PC flow imaging can possibly measure aortic blood flow helicity and vorticity which appears to be comparable to 4D flow assessment.

**HOW THIS STUDY MIGHT AFFECT RESEARCH, PRACTICE OR POLICY**

- Helicity and vorticity can possibly be quantified with 2D PC CMR, potentially allowing retrospective studies using large databases to infer population-level disease association of aortic flow haemodynamics in several cardiovascular diseases. This will enable not only simple clinical translation but also future studies to investigate the prognostic role of aortic flow using large databases.

## Introduction

Aortic blood flow exhibits a complex and distinctive multidirectional pattern, influenced by the structure of the aortic valve, the shape and branching of the ascending aorta, as well as the compliance and elasticity of the aortic wall. Healthy individuals’ aortic blood flow has a characteristic spiral component defined as helicity along with a predominant laminar flow.^1–3^ Aortic vortical flow is a swirling or rotational movement of blood within the aorta which has been linked to increased wall stress, potentially leading to atherosclerosis, thrombus formation, increased wall stiffness and accelerating hypertension.^4 5^ Factors such as left ventricular function, aortic valve disease, aortic compliance and dilatation impact the helicity and vorticity of aortic blood flow. This could possibly impact aortic conduit and reservoir functions.^6 7^

Traditionally, quantitative blood flow analysis has been based on ECG-gated time-resolved two-dimensional (2D) phase contrast (PC) cardiovascular MR (CMR) for peak velocity, regurgitation fraction, stroke volume and shunt volumes.^8 9^ There are certain limitations with this modality, mainly to do with single directional velocity encoding, which does not provide direct information about helicity or vorticity of blood flow. four-dimensional (4D) flow CMR, on the other hand, allows comprehensive visual assessment and obtains blood flow analysis along three spatial dimensions, three velocity directions and time in a cardiac cycle.^10 11^ Functional haemodynamic parameters, such as helicity, vorticity and flow displacement (FD), can be derived retrospectively at any location within the thoracic aorta.^12–16^ However, 4D flow CMR remains complex, requiring long acquisition time and postprocessing, and, more importantly, is not widely accessible.

As such, despite some of the advances in knowledge and technology for in vivo assessment, there is limited literature on assessing these functional parameters of aortic flow and their impact on clinical outcomes in various cardiovascular diseases. For broader clinical assessment of helical and vortical flows in the aorta and their clinical role investigation, simplified approaches are needed, which can be measured by both 2D PC and also by 4D flow CMR. This will allow not only simple clinical translation but also future studies to investigate the prognostic role of aortic flow using large databases. Therefore, the main objective of this study was to develop and validate pragmatic 2D PC methods to quantify both helicity and vorticity of blood flow in the aortic root.

## Methods

### Study cohort

For this proof-of-concept study, we identified patients from the PREFER-CMR registry (ClinicalTrials.gov: NCT05114785). We enrolled five cases of each aortic stenosis (AS) and heart failure with preserved ejection fraction (HFpEF) categories. The main inclusion criteria for patients were over 18 years of age and a confirmed diagnosis of AS or HFpEF by CMR. We selected HFpEF and AS patient groups because both these diseases are associated with aortic pathology and possible flow disturbances.^17 18^ The exclusion criteria were limited to any CMR contraindication. We also included five healthy controls (HCs) from previous research registries in this study. The main inclusion criteria for the HCs were >18 years of age and no prior history of cardiovascular disease.

### Ethics approval and consent to participate

This study was conducted according to the principles outlined in the Declaration of Helsinki—Version 2013. The collection and management of data were approved by the National Research Ethics Service in the UK (21/NE/0149). A pragmatic opt-out informed consent was obtained from all patients included in the study.^19^

### CMR protocol

CMR study was performed on a 1.5 Tesla Magnetom Sola Siemens system with a superconducting magnet (Siemens Healthineers AG, Erlangen, Germany). All patients were examined in the supine position, headfirst, using a respiratory sensor and ECG gating. Additionally, the scanner was equipped with an 18-channel biometric body coil.

The CMR protocol included baseline survey images and cines, gadolinium enhancement imaging and 4DF acquisition methods previously described by our group.^20–25^

For standard cines, we acquired 30 phases throughout the cardiac cycle. Other cine acquisition parameters include TR: 2.71, TE: 1.13, field of view (FOV): 360×289.3 mm^2^ with Phase FOV—80.4%, number of signal averages (NSA): 1, matrix: 224×180 (phase), bandwidth: 167.4 kHz, (930 Hz/Px), flip angle: 80, slice thickness: 8 mm and Grappa acceleration with a factor of 2.

For 4D flow acquisition, the initial velocity-encoding value (VENC) setting was 150–200 cm/s for all HCs and HFpEF cases. For patients with AS, the initial VENC was 400 cm/s. For 4DF, we acquired 30 phases throughout the cardiac cycle to keep the data consistent with cines. The acquired temporal resolution was 40 ms. Other 4D flow acquisition parameters include TR: 4.98, TE: 2.71, field of view (FOV): 200×256.3 mm^2^, NSA: 1, acquired voxel size=3×3×3 mm^3^, bandwidth: 31.616 kHz, (494 Hz/Px), flip angle: 5 and Grappa acceleration in the phase-encoding direction with a factor of 2 and slide direction of 1. The ECG was retrospectively gated with free breathing to avoid diastolic temporal blurring.

### Image analysis

**Figure 1:**
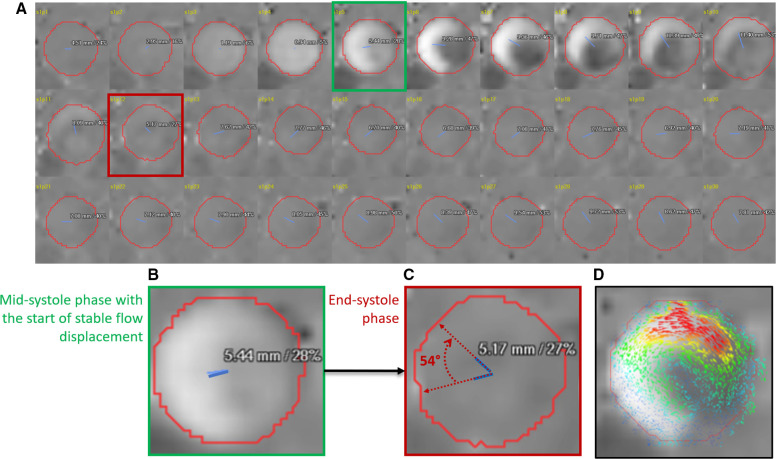
(A) Time-resolved 30 passed of two-dimensional (2D) phase contrast through-plane velocity mapping demonstrating systolic flow displacement (FD) (blue line from the centreline of the aorta at the mid-main pulmonary artery level) rotation during systolic phases. (B) The first rotational angle was recorded after the peak systole when the FDs is greater than 12% and has a stable value (green phase). (C) The second recorded phase to compute the rotational angle is at the end of the systole (red phase). (D) Concomitant, superimposed 2D vector using 4D flow CMR data was used to appreciate the direction of rotation when comparing it with the direction of rotation using FDs. CMR, cardiovascular MR.

All image analyses were postprocessed with the in-house developed MASS research software (MASS; Version 2022-EXP, Leiden University Medical Center, Leiden, the Netherlands). A static reformatted plane was planned through the ascending aorta at the mid-main pulmonary artery level to generate a through-plane velocity encoded 2D PC data using 4D flow CMR data. This plane was treated as a 2D PC plane. Ascending aortic helical flow was defined as the flow swirling around the aortic centreline. Ascending aorta vortex flow was defined as any flow rotating on the long axis of the aorta near the inner curvature of the aortic root^26^ (figure 1).

### Bench testing and manual assessment

#### FD and its threshold

First, we developed methods to superimpose the FD angle on top of the 2D PC image for each phase in the cardiac cycle. FD was calculated using published methods.^7^ FD was calculated as the distance between the vessel centreline and the centre of the eccentric flow and was normalised for overall vessel size. It is presented as a percentage in this paper. In bench-testing, significant noise was noted in systolic FDs due to mainly minimal FD when there was mainly laminar flow in early systole. Importantly, in early systole, laminar flow resulted in FD being lower than 12% and rotating around randomly without any meaningful flow rotation. Hence, we used an FD threshold of 12% to circumvent this issue for all calculations. Additionally, we recorded FD in late systole (FDls) and diastole (FDd) using the same methods.

#### Manual tracking of rotational flow angle

**Figure 2:**
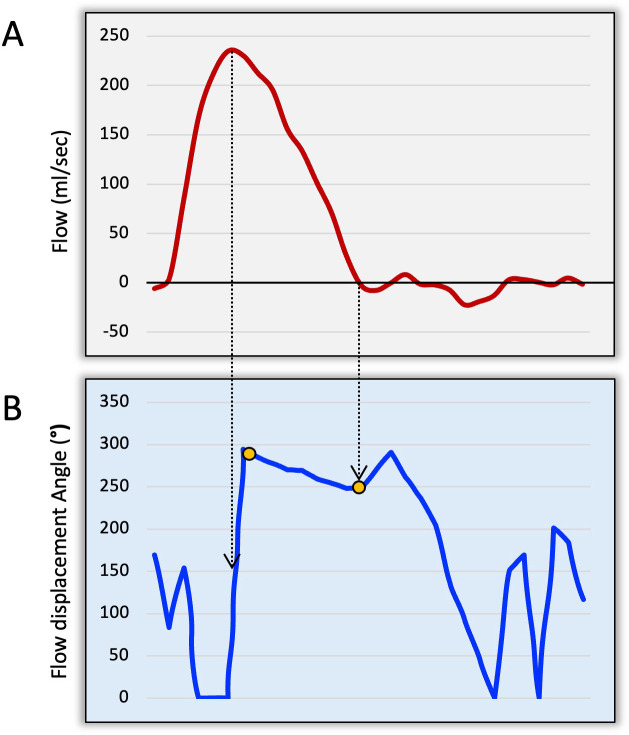
(A) Aortic flow curve to identify peak systolic and end-systolic phases. (B) Rotational angle curve demonstrating where the start and end were recorded (orange dots). The start was recorded after normalisation from the baseline.

The rotational flow was manually determined by mapping the angle of movement of FD from the peak systolic phase (FDs_peak_) till the end of systole (figure 1). To better appreciate the rotational flow and its direction, we used superimposed 2D vectors using 4D flow datasets. We then calculated the systolic rotational flow angle change (ΔsRA) from mid-systole till the end of systole (figure 2). The rotational direction was recorded as clockwise or counterclockwise (figure 1).

#### Manual mapping of vortical flow

**Figure 3:**
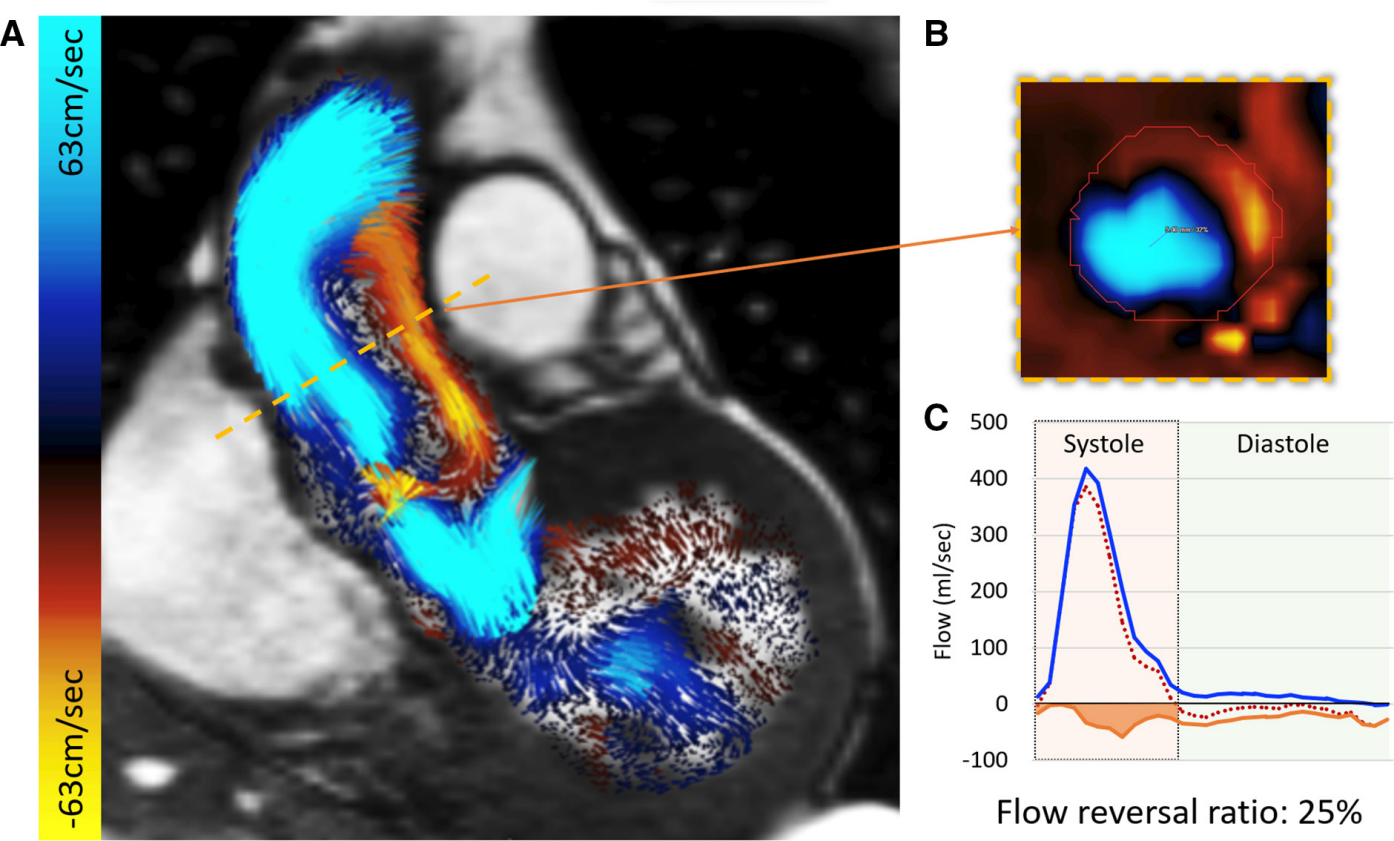
(A) Vortical flow was visually identified in the long-axis aortic root views. During systole, at mid-main pulmonary artery level, both forward (blue) and backward (orange/red) flows are observed. (B) A reformatted cross-sectional phase contrast through plane demonstrating both forward and backward flows associated with longitudinal vorticity of blood flow near the inner curvature of the ascending aortic root. (C) Flow curves demonstrating the significant amount of backward flow compromising aortic conduit function.

Manual qualitative assessment of longitudinal vortical flow in the ascending aorta during systole was done using coronal cine with superimposed 2D velocity vectors with Doppler colour-coded signals of forward and backward flow. If, on visual assessment, significant vortical flow was noted, it was recorded (figure 3).

#### Automated systolic rotational angle change

This was done without any visual input and solely using the rotational angle (RA) time-resolved curve of the FD line. Peak systolic phase was automatically registered as the peak flow rate on the flow curve. End systolic phase was determined where the downward slope of the descending systolic flow curve intersected with the x-axis (or no flow). Systolic RA change was determined from the point the flow angle stabilised after peak systole till the end of systole on the RA curve (figure 2).

#### Automated flow reversal ratio

Flow reversal ratio (FRR), systolic forward and systolic retrograde flows (SRF) were calculated as previously described in the literature.^27^ However, in this study, we quantified it in the total cardiac cycle and in the systolic phase only (sFRR)—from the start to the end of systole.

The equation to calculate the FRR was as follows:

$$\mathrm{FRR}(\%)=\left|Q_{\text{retrograde}}\left(t_{\text{systole}}\right)\right|/Q_{\text{forward}}\left(t_{\text{systole}}\right)\times 100$$

where Q_forward_ (t_systole_) and Q_retrograde_ (t_systole_) represent the forward and retrograde flows at peak systole flow.^27^

All CMR contour tracing and 4D flow analysis were performed by an EACVI level-III certified CMR expert (PG) with over 10 years of CMR experience and academic fellows (ZM and HA) under direct supervision. HA (3 years experience in advanced CMR) repeated the analysis for all cases using the same postprocessing protocol for reproducibility testing.

### Statistical analysis

Data analyses were performed by using MedCalc (MedCalc Software, Ostend, Belgium, V.20.215). Data were treated as non-parametric due to small numbers. Continuous variables are expressed as median±IQR. Mann-Whitney U test was used to compare the independent variables. Agreement between visual and quantitative assessments and reproducibility analyses was done using several methods: Pearson’s correlation coefficient (r), Bland-Altman plots intraclass correlation coefficient (ICC) and C-statistics.

For comparing manual versus automated ΔsRA, we used Pearson’s correlation coefficient to assess the correlation and Bland-Altman plots to assess bias and agreement. For comparing categorical vortex visual analysis against FRR and other metrics of FD, we evaluated the area under the curve (AUC) using receiver operating characteristic curves. We used ICC to assess the correlation and Bland-Altman plots for reproducibility testing to assess bias and agreement. The significance threshold was set at p<0.05.

## Results

### Study population

Patient characteristics are summarised in table 1. A total of 15 cases (46% males) were included in this study. Of these, five were HCs, five were patients with HFpEF and five were AS patients. The median age of our cohort was 76 years (IQR: 56–81 years). The median height was 169 cm (IQR 162–178 cm) and the median weight was 81 kg (IQR 72–96 kg). Among the patients, 6 (60%) had hypertension, 60% had dyslipidaemia and 4 (40%) were diabetic. Other comorbidities included previous cerebrovascular accidents (2 (20%)) and myocardial infarction (1 (10%)). HCs were younger (52±12 years) compared with both HFpEF (80±12 years, p=0.02) and AS cohorts (78±7 years, p=0.02) with no cardiovascular risk factors.

**Table 1:** Baseline characteristics of the study population

	HC (n=5)	HFpEF (n=5)	AS (n=5)	P value*	P value†
Age (years)	52±12	80±12	78±7	0.02	0.02
Gender (male)	2 (40)	2 (40)	3 (60)	1	0.55
Height (cm)	170±18	168±13	168±20	0.75	1
Weight (kg)	76±26	86±26	74±27	0.53	1
Diabetes mellitus		2 (40)	2 (40)		1
Hypertension		4 (80)	2 (40)		0.22
Myocardial infarction		1 (20)	2 (40)		0.51
Cerebrovascular accident		2 (40)	0 (0)		0.14
Dyslipidaemia		3 (60)	3 (60)		1

All data are presented in median±IQR or n (%).

*HC versus HFpEF.

†HC versus AS.

AS, aortic stenosis; HC, healthy controls; HFpEF, heart failure with preserved ejection fraction.

### Healthy versus patients

All HCs (5 (100%)) had a clockwise rotation, while 2 (20%) of patients had an anticlockwise flow rotation, of which 1 had both rotations. Patients had a significantly higher FDs_peak_ (19%±21%) and FDs_avg_ (28.5%±7.5%) compared with HCs (all p=0.03). Similarly, other aortic flow parameters showed significant differences between patients and HCs, including FDls_avg_ (34.6%±7.7% vs 23%±11.2%, p=0.04) and SRF (15.6±14.2 mL vs 2.6±2.2 mL, p<0.0001). Moreover, healthy volunteers had statistically lower total FRR and sFRR when compared with patients (15%±14.3% vs 37.2%±13.9%) and (4.5%±3.7% vs 22.5±11.2%), subsequently. However, no statistical differences were observed between the two groups for RA (p=0.06), average diastolic FDd_avg_ (p=0.06) and systolic forward flow (p=0.22). A comparison of 2D PC derived helicity and vorticity parameters of blood flow between HCs and patients with HFpEF and AS combined is summarised in table 2.

**Table 2:** Comparison of helicity and vorticity parameters of blood flow between healthy cohorts and patients with HFpEF and AS combined

	Healthy controls	Patients	P value
Number	5	10	
Net aortic forward flow (mL)	58±28	58.4±16.9	0.86
Net aortic backward flow (mL)	1.5±5.2	6.2±4.2	0.16
Aortic maximum area (cm^2^)	9.2±3.4	11±5.9	0.09
Aortic minimum area (cm^2^)	6.6±2.2	8.6±2.8	0.13
Systolic forward flow (ml)	64±27.6	71.5±21.3	0.22
Markers of flow eccentricity			
Flow displacement in peak systole (%)	5±3	19±21	0.03
Average systolic flow displacement (%)	19±4.7	28.5±7.5	0.03
Average late systolic flow displacement (%)	23±11.2	34.6±7.7	0.04
Average diastolic flow displacement (%)	30.5±21	40.1±16.4	0.59
Markers of flow vorticity			
Systolic retrograde flow (mL)	2.6±2.2	15.6±14.2	<0.0001
Systolic flow reversal ratio (%)	4.5±3.7	22.5±11.2	<0.0001
Total flow reversal ratio (%)	15±14.3	37.2±13.9	0.01
Marker of flow helicity			
Rotational angle (°)	7±17	35±31.6	0.06
Clockwise rotation (righthand)	5 (100)	8 (80)	0.17

All data are presented as median±IQR and n (%).

AS, aortic stenosis; HFpEF, heart failure with preserved ejection fraction.

### RA agreement

**Figure 4:**
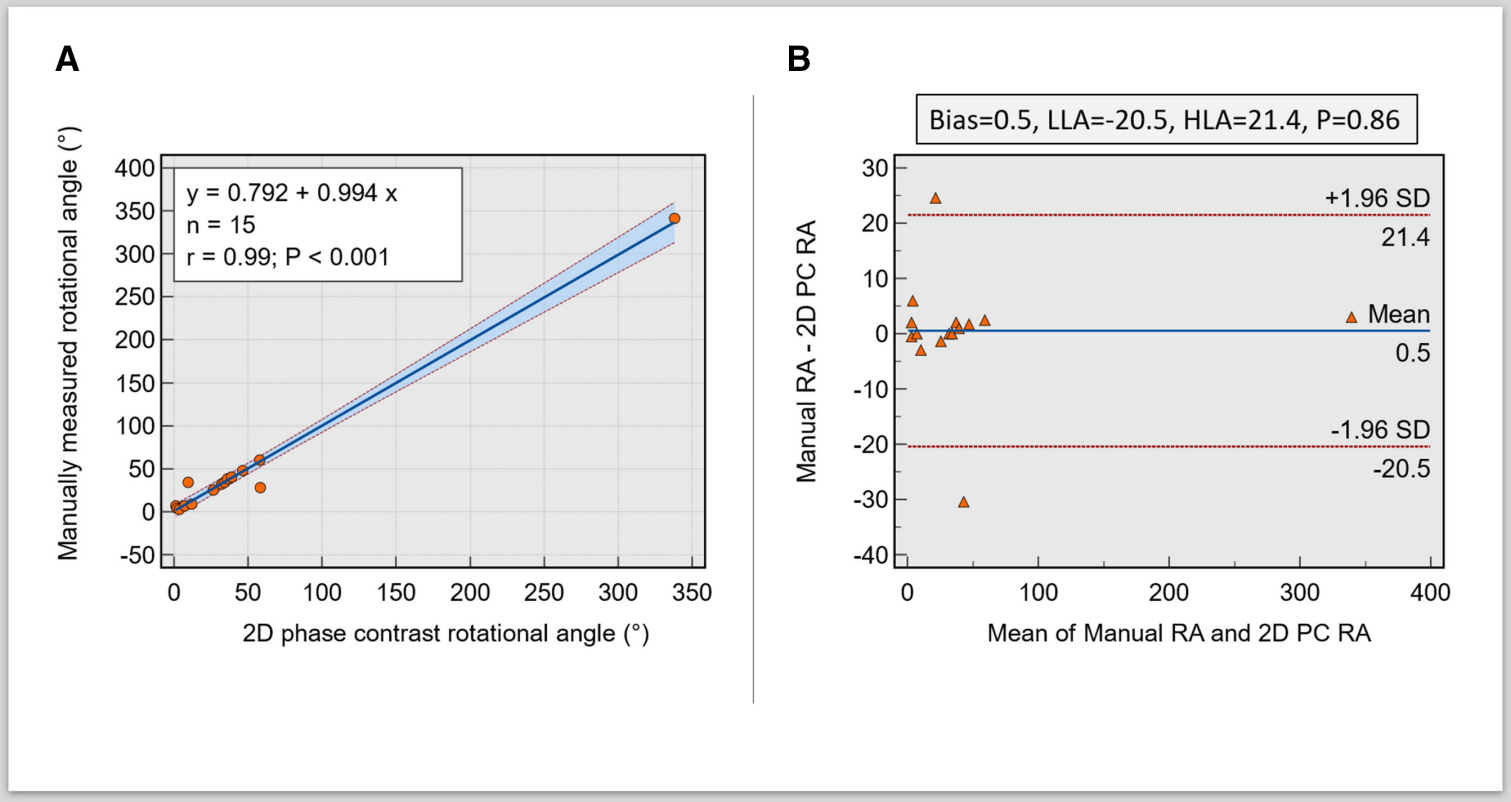
(A) Scatter plot demonstrating an excellent association between manual and automated methods of rotational angle calculation. (B) Bland-Altman plots show minimal bias between the manual and automated rotational angles from the curve. 2D, two dimensions; PC, phase contrast; RA, rotational angle; LLA, lower limit of agreement; HLA, higer limit of agreement.

Manual ΔsRA demonstrated excellent association with semiautomated ΔsRA (r=0.99, 95% CI 0.9907 to 0.999, p<0.001). In addition, there was an excellent agreement with semiautomated ΔsRA (bias=0.5, limits of agreement=−20.5 to 21.4, p=0.86) (figure 4).

The agreement between manual assessment of rotational direction by investigation of 4D flow vectors and computed direction was in good agreement (kappa=0.76; 95% CI 0.32 to 1).

### Vortical flow associations

As seen in table 3, patients with vortical flow had higher FDs_avg_ (p=0.03), FDls_avg_ (p=0.02) and FDd_avg_ (p=0.02). Furthermore, there was a significant difference between patients with no vortex present and patients with vortex for SRF (2.8±2.2 mL vs 14.9±16.1 mL, p=0.02), sFRR (3.8%±3.6% vs 20.5%±15%, p=0.01) and total FRR (16.9%±12.1% vs 37.1%±13.6%, p=0.02). Patients with vortical flow had a larger aortic maximum (11.2±3.8 cm^2^) and minimum areas (8.7±2.5 cm^2^) (p=0.04 and p=0.03, respectively). The two groups had no statistical differences in RA and systolic and net forward flows.

**Table 3:** Comparison of helicity and vorticity parameters of blood flow between patients with and without vorticity

	No vortex present	Vortex present	P value
Number	4	11	
Net aortic forward flow (mL)	60.5±26.1	58±18.9	0.85
Net aortic backward flow (mL)	2.1±5.6	5.7±4.6	0.34
Average systolic flow displacement (%)	18.5±3.9	28.3±7.4	0.03
Average late systolic flow displacement (%)	20.5±7.2	34.3±7.3	0.02
Average diastolic flow displacement (%)	25.7±6.3	42±11.9	0.02
Rotational angle (°)	10.7±20.3	34±43.2	0.28
Systolic forward flow (mL)	64.5±23.4	71.3±18.1	0.47
Systolic retrograde flow (mL)	2.8±2.2	14.9±16.1	0.02
Systolic flow reversal ratio (%)	3.8±3.6	20.5±15	0.01
Total flow reversal ratio (%)	16.9±12.1	37.1±13.6	0.02
Aortic maximum area (cm^2^)	9.4±3.4	11.2±3.8	0.04
Aortic minimum area (cm^2^)	6.5±2.1	8.7±2.5	0.03

All data are presented as median±IQR and n (%).

### Vortical flow agreement

**Figure 5:**
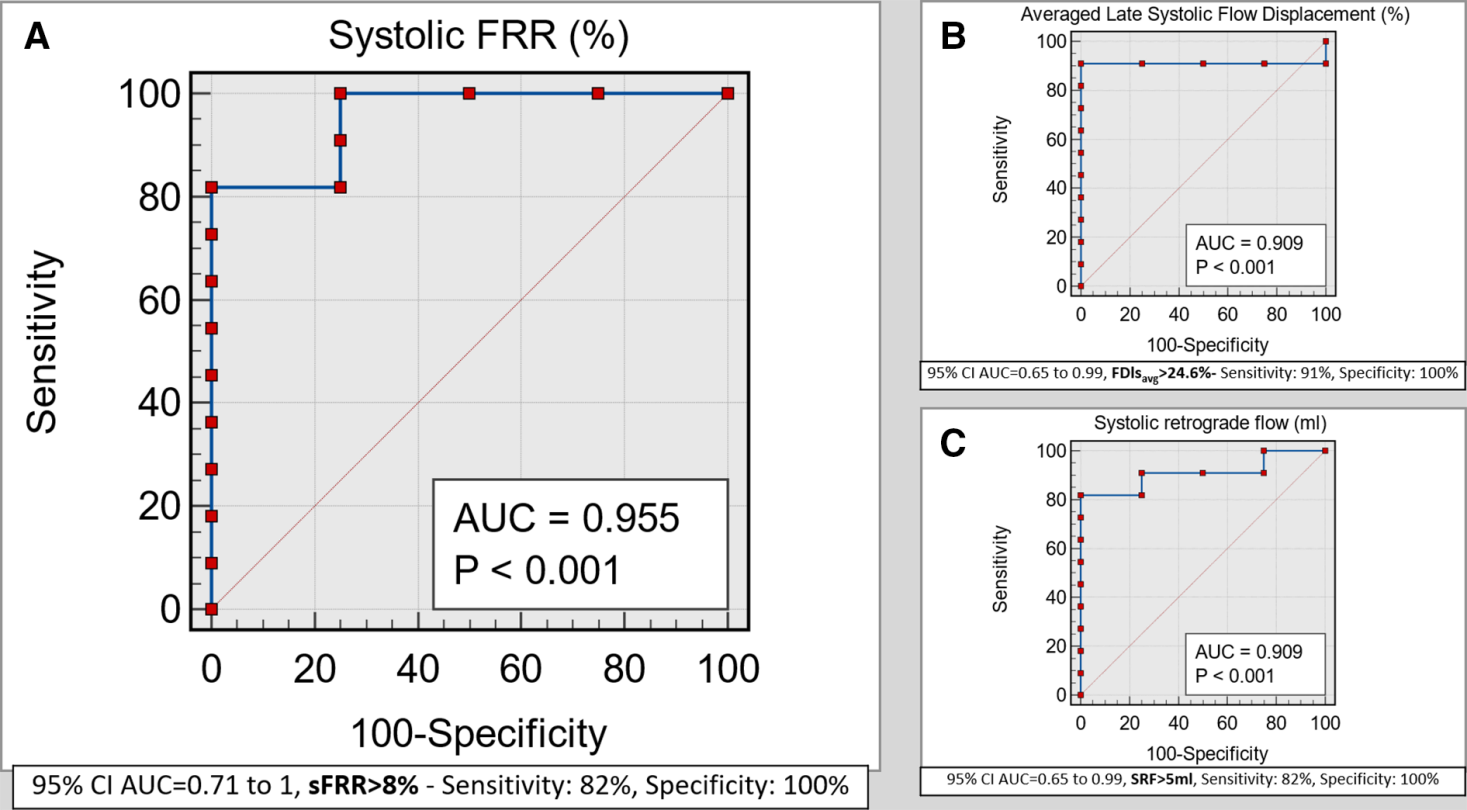
(A) Receiver operator curve demonstrating excellent agreement between visual assessment of longitudinal vortex flow in ascending aorta and systolic flow reversal ratio (sFRR) percentage. (B, C) Receiver operator curve showing good correlation between visual assessment of longitudinal vortex flow in ascending aorta and averaged flow displacements in diastolic phases and also the retrograde systolic flow, which is happening directly as a result of the vortex. AUC, area under the curve.

The FDls_avg_, a marker of flow eccentricity, demonstrated an AUC of 0.909, p<0.001 for visual vortex flow presence near the inner curvature of ascending aortic root. A threshold of >24.6% for FDls_avg_ had a sensitivity of 91% and a specificity of 100% for visual vortex presence (figure 5). Similarly, SRF demonstrated an AUC of 0.909, p<0.001 for visual vortex flow presence near the inner curvature of the ascending aortic root. A threshold of >5 mL for SRF had a sensitivity of 82% and specificity of 100% for visual vortex presence (figure 5).

However, sFRR demonstrated an AUC of 0.955, p<0.001 for visual vortex flow presence near the inner curvature of ascending aortic root. A threshold of >8% for sFRR had a sensitivity of 82% and specificity of 100% for visual vortex presence (figure 5).

### Reproducibility

Measures of interobserver variability are provided in table 4. The intraclass correlation between all parameters remained good (between 0.87 and 0.99). The bias for flow indices was minimal (0–4 mL). FDs_avg_ showed a minimal bias at 1.28% with a high ICC of 0.92. Similarly, sFRR had a minimal bias of 1.14% with an ICC of 0.96.

**Table 4:** Interobserver repeatability analysis for vortical and helical flow parameters by 2D PC CMR

	Bias	ICC	P value
Net aortic forward flow (mL)	1.84	0.98	0.57
Net aortic backward flow (mL)	0.53	0.97	0.35
Average systolic flow displacement (%)	1.28	0.92	0.45
Average late systolic flow displacement (%)	2.01	0.90	0.44
Average diastolic flow displacement (%)	3.43	0.87	0.36
Rotational angle (°)	5.72	0.99	0.5
Systolic forward flow (mL)	3.19	0.99	0.26
Systolic retrograde flow (mL)	2.28	0.95	0.25
Systolic flow reversal ratio (%)	1.14	0.96	0.33
Total flow reversal ratio (%)	0.99	0.95	0.39
Aortic maximum area (cm^2^)	0.92	0.94	0.17
Aortic minimum area (cm^2^)	0.64	0.89	0.20

Bias has been computed using Bland-Altman plot analysis. Absolute values of bias are presented.

CMR, cardiovascular MR; 2D, two dimensions; ICC, intraclass correlation coefficient; PC, phase contrast.

## Discussion

In this study, we demonstrated that both helicity and vorticity can possibly be quantified using 2D PC CMR. Change in systolic RA change (ΔsRA), a marker of helicity of blood flow, demonstrated excellent agreement between manual and semiautomated methods, yielding a Pearson’s correlation coefficient of 0.99 and a bias of 0.95. In addition, sFRR demonstrated excellent diagnostic agreement with visual assessment of systolic vortical flow around the inner curvature of the aortic root. Even diastolic FD, a marker of flow eccentricity and SRF were associated with vortical flow in the ascending aortic root.

### Systolic flow reversal: a marker of vorticity

Systolic flow should be predominantly anterograde, with any retrograde flow impairing aortic root conduit function, as captured by an increase in FRR. The relevance of FRR has been shown in patients with bicuspid aortic valve (BAV)^27^ and AS previously.^28^ Barker *et al* 27 demonstrated that all BAV patients with AS had FRR >10%. Our study did not investigate BAV, but in all patients with HFpEF and AS, we noted similar higher systolic FRR with a median value of 22.5%. Ha *et al* 28 retrospectively analysed haemodynamic characteristics of ascending aorta in 80 patients with severe AS using 2D PC CMR and showed significantly higher FRR in patients with aortic root dilatation (>4 cm). The study also showed that subsequent aortic dilatation was associated with higher aortic flow skewness. The major strength of these studies is that they did not use 4D flow CMR and demonstrated the clinical relevance of aortic flow haemodynamics assessed by 2D PC only.

Nevertheless, even though it is plausibly clear that physiological FRR should correspond to vortical flow in the ascending root, a clear threshold has yet to be established. This study utilises 4D Flow CMR for qualitatively and quantitatively validating these aortic physiological flow parameters to 2D PC methods. Importantly, we showed that an sFRR cut-off of >8% was 82% sensitive and 100% specific for visually present vortex during systole. This adds to the growing body of evidence that increased FRR is plausibly associated with reduced aortic conduit function and should be further investigated as a potential metric for improving risk stratification of patients in aortopathy, aortic valvulopathy and other cardiovascular conditions.

### Systolic RA change: a marker of helical flow

A study by Frydrychowicz *et al* demonstrated aortic flow to be predominantly right-handed helical flow by 4D flow CMR streamline visualisation in 62 healthy subjects.^29^ These findings are consistent with our manual and semiautomated analysis and in agreement with clockwise rotation in 2D. Previous studies have investigated ascending aortic helical flow by three-dimensional (3D) streamlines using 4D flow CMR and have shown that a rotation of <180° is considered normal.^30 31^ The clinical significance of the increased helical flow remains poorly understood. Physiologically, it is plausible to speculate that a significant increase in helicity of the blood flow in the aorta will increase the overall distance the blood needs to travel during systole and, hence, compromise systolic force in the periphery. This could affect distal perfusion and potentially cause ischaemia. Bürk *et al* have shown that helical aortic flow increases in patients with a dilated ascending aorta^31^ or aortic valve disease.^32^ Even though our study is not directly quantifying the same angle in 3D of the streamlines but instead is quantifying the time-resolved RA in a 2D through-plane, we have demonstrated a similar finding that helicity of blood flow increases in patients not only with AS but also with HFpEF. However, the reference range of the systolic RA change (ΔsRA) investigated in our study remains to be determined in healthy populations. The methods we propose can potentially allow broader clinical translation and investigation of both physiological and pathological aortic flow in several cardiovascular diseases.

### Limitations

There are several limitations to the present study. First, the number of controls and patients is insufficient to infer any population-level disease association with flow patterns and their quantification. Second, we have used a different method to validate the helicity of blood flow compared with what has been used in the literature before. The main reason for doing so was to use a time-resolved approach and have better agreement with the rotation in 2D. Third, this study only tests complex flow haemodynamics at the level of ascending aorta (mid-main pulmonary artery level). Hence, the results of this work should be cautiously judged at other levels in the aortic root and other large vessels, for example, the main pulmonary artery. Finally, our work did not evaluate these methods in bicuspid aortic valve patients, and future studies need to evaluate how reliable these methods are in that patient population.

## Conclusion

3D PC flow imaging can possibly quantify blood flow helicity (ΔRA) and vorticity (FRR). These imaging biomarkers of flow helicity and vorticity demonstrate high reproducibility for clinical adoption. Further research is needed to validate these findings in larger, more diverse patient populations.

## Data Availability

Data are available on reasonable request. The datasets generated and analysed during the current study are not publicly available. Access to the raw images of patients is not permitted since specialised postprocessing imaging-based solutions can identify the study patients in the future. Data are available from the corresponding author on reasonable request.
